# Food safety and security: what were favourite topics for research in the last decade?

**Published:** 2011-06

**Authors:** Ana Marušić

**Affiliations:** Croatian Centre for Global Health and Department of Research in Medicine and Health, University of Split School of Medicine, Split, Croatia

## Abstract

The world is faced with the challenge to feed an estimated 9 billion population of the Earth by 2050. To address the scientific evidence for the safety of food, I searched the Web of Science bibliographical and citation database for most cited articles from this research area. The topics with greatest impact on the research community, judged by their annual rate of citations during the last decade, were food-borne pathogens and toxins, with emerging genetic studies and new methods of visualising toxins on surfaces. Epidemiological and survey studies demonstrated that there was systematic effort to document, rapidly detect and control epidemic spread of disease and that these measures decreased the threat to food safety in developed countries, but that there is still much room for improvement. Research relevant for developing countries included the potential molecular targets to alleviate accumulation of arsenic in rice. As in other areas of research and life, human factor seems to be the most important one for the safety of food. The five keys to safer food of the WHO – keep clean, separate raw and cooked, cook thoroughly, keep food at safe temperatures, use safe water and raw materials – are thus still very relevant for the developed as much as the developing world.

The safety of food is an important health, social and economical issue. According to the World Health Organization (WHO), foodborne and waterborne diarrhoeal diseases kill an estimated 2.2 million people annually, 1.9 million of them children (1). Unsafe food can be the cause of or contribute to many diseases, from diarrhoea to some cancers, so that food safety, nutrition and food security are among WHO’s 13 strategic objectives (1). Food safety also has potential impact on at least 4 of the 8 millennium development goals set by the United Nations for 2015 (2): eradication of extreme poverty and hunger, reduction of child mortality, improvement of maternal health, and ensuring of environmental stability. To ensure safer food for health, WHO also developed training materials called ‘Five Keys to Safer Food’, promoting simple health measures based on evidence from scientific research for use of food handlers, including customers, in order to decrease the burden of foodborne diseases (3).

Food and its safety has become the topic of globally increasing research efforts, particularly in view of the growth of human population. The interest of the scientific community in food safety is illustrated by the recent special issue of the *Science* magazine, which explored the potential of science to tackle the challenge of feeding the estimated 9 billion people who will inhabit the Earth by 2050 (4). The topic stirred a heated debate on the printed and electronic pages of the journal (5). The 2011 crisis at a nuclear power plant after the earthquake in Japan and the detection of radioactivity in certain food samples contributed to the concerns about the safety of food from that area (6). Most recent outbreak of a deadly haemolytic-uremic syndrome in Germany, caused by bacterial contamination of vegetable sprouts (7), also drew the attention to food safety. In view of the attention of the scientific community to the topic of food, I was interested in the scientific evidence for its safety. To assess research published on this topic in the last decade, I searched the Web of Science, bibliographical database that also uses citations to published research as a measure of impact on research community (8).

## METHODS

I performed the search of the *Web of Science* (WoS), the citation database of the Thomson Reuters, formerly the Institute for Science Information (ISI) (8). WoS was chosen as a widely used citation database (9-12), so that the results of the study could be comparable to other citation analyses. The search was performed on 18 March 2011 and included all databases available in WoS (*Science Citation Index Expanded*, *Social Sciences Citation Index* and *Arts and Humanities Citation Index*). The search term was ‘food safety’, as this term is used by the WHO (3) and the time span was limited to the last 10 years (2001–2010). The search was then refined by selecting ‘article’ as the document type. The articles with highest citations rates, defined as the number of citations per year after publication (11), were analyzed. WoS tools were used to present the number of articles and their citations, relevant research areas, leading journals, countries, institutions and funding sources.

To get an insight into the possibly most influential articles in the area of food safety, I identified top 10 articles according to their citation intensity, defined as the average number of citations received per year after the publication date. Because of the different times of publication, the total number of citations at a certain time point may not be the best measure of the article’s visibility and influence, so the citation intensity was taken as the proxy for the interest of the research community for the research, regardless of the time of its publication (11). Only the most recent studies, particularly those published in 2010 would have disadvantaged by such approach (11).

## RESULTS AND DISCUSSION

**Figure 1:**
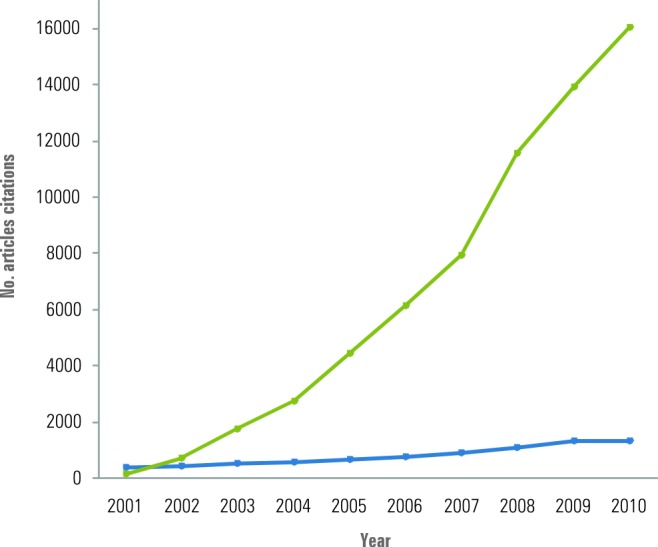
Number of publications about food safety (blue line), classified as ‘articles’ by the *Web of Science*, and citations to these publications (green line) in 2001–2010.

The search retrieved 11 565 out of 14 417 309 indexed items for the 2001–2010 time span. Out of those, 69.6% were designated as ‘articles’ (n=8044) and the rest were ‘reviews’ (13.1% items) ‘proceedings papers’ (10.5%), or other types of bibliographical items (**Table 1**). Items classified as ‘articles’ by WoS should bring results of original research (11), so that further analysis was performed only for this bibliographical item. As the database retrieved articles and/or citations to some items from 2000 and 2011, citation data for individual articles were manually checked and only the relevant post-publications years up to the end of 2010 were included in the analysis. Descriptive data on the total publications (**Table 1** and **Figure 1**) were presented for all retrieved items because it was not possible to separate citations for outlying years.

**Table 1:** Types of items, areas of research and top 10 countries, funding agencies, institution and journals publishing research on food safety in 2001-2010*

Bibliographical characteristic	No.	%†
Type of item published (n = 11565):		
article	8044	69.6
review	1512	13.1
proceedings paper	1215	10.5
editorial material	385	3.3
meeting abstract	182	1.6
news items	162	1.4
letter	35	0.3
book review	16	0.1
correction	9	0.08
reprint	3	0.03
bibliographical item	1	0.01
software item	1	0.01

Areas of research (articles only, n = 8044):		
food science & technology	1547	
biotechnology & applied microbiology	501	
nutrition & dietetics	473	
veterinary sciences	368	
environmental sciences	360	
public, environmental & occupational health	332	
microbiology	330	
agriculture, multidisciplinary	329	
economics	278	
chemistry, applied	211	

Countries (articles only):		
USA	3057	38.0
England	545	6.8
Germany	496	6.2
Canada	415	5.2
Italy	390	4.8
France	385	4.8
Spain	369	4.6
Peoples R. of China	355	4.4
Netherlands	347	4.3
Japan	286	3.6

Funding agencies (articles only, n = 8044):		
European Commission or European Union	62	0.8
National Natural Science Foundation of China	34	0.4
National Institutes of Health	30	0.4
Ministry of Agriculture of the Czech Republic	14	0.2
National Science Foundation	12	0.1
United States Department of Agriculture	12	0.1
Ministry of Education, Youth and Sports of the Czech Republic	11	0.1
National Council for Scientific and Technological Development (CNPq), Brazil	9	0.1
Pfizer	9	0.1
Public Health Agency of Canada	6	0.1

Institutions (articles only, n = 8044):		
US Department of Agriculture, Agriculture Research Service	143	1.8
US Department of Agriculture	89	1.1
Wageningen University & Research Centre, The Netherlands	74	0.9
Cornell University, USA	70	0.9
Ohio State University, USA	64	0.8
Michigan State University, USA	63	0.8
Chinese Academy of Sciences	62	0.8
University of Guelph, Canada	60	0.7
University of California Davis, USA	58	0.7
Institut Scientifique de Recherche Agronomique, France	53	0.7

Journals (articles only, n = 8044):		
*Journal of Food Protection*	336	4.2
*Food Control*	216	2.7
*Food and Chemical Toxicology*	193	2.4
*International Journal of Food Microbiology*	188	2.3
*Journal of Agricultural and Food Chemistry*	141	1.8
*British Food Journal*	97	1.2
*Journal of Food Science*	84	1.0
*Regulatory Toxicology and Pharmacology*	83	1.0
*Annals of Pharmacotherapy*	74	0.9
*Journal of Applied Microbiology*	73	0.9

Languages (articles only, n = 8044):		
English	7481	93.0
German	205	2.5
French	89	1.1
Spanish	51	0.6
Portugese	49	0.6
Chinese	33	0.4
Japanese	33	0.4
Hungarian	28	0.3
Polish	21	0.3
Korean	18	0.2

*Data from *Web of Science* (WoS), search performed 18 March 2011. Categorisation of items is according to WoS.

†Percentages were calculated from total number of published items in 2001-2010 (n=11 565) for the type of bibliographical item or the total number of articles (n=8044) for all other items. Percentages for areas of research were not calculated as articles may be assigned to more than 1 area of research.

### Time trends in food safety research publications

The number of articles continually increased over time, from 385 in 2001 to 1316 in 2009 and 1307 in 2010 (**Figure 1**). These articles received increasing numbers of citation over the years, from 128 in 2000 to 16 018 in 2010 (**Figure 1**). The number of citations increased more rapidly than the number of published articles, demonstrating the growing interest for and the impact of food safety research. The causes for the increasing trend are not clear, and may include a number of factors, from the increasing number of relevant journals covered by the database; growing number of researchers in this area; increased interest of funders, both public and commercial; increased collaboration in the field, particularly in globally relevant topics, or improved quality of research which generates more and better (and more publishable) data. While it is difficult to assess factors related to the research community, the journal coverage of the Thompson Reuters’ databases increased 22% from 2002 to 2010 (12) and surely contributed to the general increase in publications and their citations.

Most of the published articles were classified into the category of ‘Food science & technology’, followed by a range of related categories, from ‘Biotechnology and applied microbiology’ to ‘Chemistry, applied’ in the top 10 categories (**Table 1**). Top 10 countries that published most articles in food safety were responsible for 82.6% of all retrieved articles (**Table 1**). Among them the leader was the USA, followed by England, Germany and Canada. The only developing country that made a significant contribution to this area was China, which may reflect the rising concerns in China over food safety, particularly after the 2008 scandal of milk formulas tainted with melamine (13).Agencies for funding research on food safety were few (**Table 1**). As most of the published articles did not carry statements on funding, it is difficult to make an objective conclusion on the extent of financial support for food safety research and the interpretation is possible only for those articles that carried funding declaration. The top 10 funding agencies provided support for only 199 of the 8044 articles (2.5%) (**Table 1**). Among them, the European Commission or the European Union funded most articles (62 articles), followed by the National Natural Science Foundation of China (34 articles).

The top 10 institutions with most published articles were responsible for 9.1% of all publications (**Table 1**). Among them, 6 were US-based, and non-US based institutions were located in The Netherlands, China, Canada and France (**Table 1**).

There was no dominating journal among the 10 journals with the highest volume of articles on food safety, which published 18.5% of all retrieved articles (**Table 1**). The lead was taken by the *Journal of Food Protection*, which published 336 articles or 4.2% of all retrieved articles. Finally, the dominating language of the publications was English (93.0% of all retrieved articles), followed by German and French (3.6%). All other languages were used by only 3.4% of the retrieved articles.

### Most cited publications on food safety

Among the top 10 articles with highest citation intensity there were 3 review articles (14-16) and 6 original research articles (17-23) (**Table 2**).

**Table 2:** Top 10 articles published in 2000-2009 with highest number of citations intensity, presented as the average number of citations per each year after publication*

Rank	Authors	Title	Bibliographical reference	Cumulative citations	Citations per year
1	Machida M, Asai K, Sano M, et al.	Genome sequencing and analysis of *Aspergillus oryzae*	*Nature* **2005**;438:1157-1161	266	44.3
2	Jarup L	Hazards of heavy metal contamination	*British Medical Bulletin* **2003**;68:167-182	236	33.7
3	Ma JF, Yamaji N, Mitani N, et al.	Transporters of arsenite in rice and their role in arsenic accumulation in rice grain	*Proceedings of the National Academy of Sciences of the USA* **2008**;105:9931-9935	80	26.7
4	Li JF, Huang YF, Ding Y, et al.	Shell-isolated nanoparticle-enhanced Raman spectroscopy	*Nature* **2010**;464:392-395	21	21.0
5	van Boekel MAJS	On the use of the Weibull model to describe thermal inactivation of microbial vegetative cells	*International journal of Food Microbiology* **2002**;74:139-159	145	16.1
6	Koopmans M, Duizer E	Foodborne viruses: an emerging problem	*International Journal of Food Microbiology* **2004**;90:23-41	128	16.0
7	Tompkin RB	Control of *Listeria monocytogenes* in the food-processing environment	*Journal of Food Protection* **2002**;65:709-725	141	15.7
8	Adak GK, Long SM, O'Brien SJ	Trends in indigenous foodborne disease and deaths, England and Wales: 1992 to 2000	*Gut* **2002**;67:832-841	125	15.6
9	Bocio A, Llobet JM, Domingo JL, et al.	Polybrominated diphenyl ethers (PBDEs) in foodstuffs: Human exposure through the diet	*Journal of Agricultural and Food Chemistry* **2003**;51:3191-3195	120	15.0
10	Zhao CW, Ge BL, De Villena J, et al.	Prevalence of *Campylobacter spp*., *Escherichia coli*, and *Salmonella* serovars in retail chicken, turkey, pork, and beef from the Greater Washington, DC, area	*Applied and Environmental Microbiology* **2001**;67:5431-5436	130	13.0

*Citations were calculated for the years after publication, including the year of publication, up to the end of 2010. The exception was the article by Koopmans and Duizer, which had 1 citation in 2003 although the official paper publication was in 2004.

The review article with the highest citation rate (34 citations per year) was published in the *British Medical Bulletin* in 2003 and addressed the hazards of heavy metal contamination, predominantly lead, cadmium, mercury and arsenic (14). Cadmium exposure comes mainly from re-chargeable nickel-cadmium batteries, which are often thrown away with the regular garbage, as well as from cigarette smoke. Exposure to mercury occurs via food, mainly fish, while there is no evidence so far that amalgam dental fillings contribute to mercury exposure and poisoning. Lead exposure currently comes primarily from emissions of petrol combustion in vehicles, while the lead-based paints and food containers have been abandoned. Finally, food is the most important source of arsenic poisoning for most populations, although drinking water could be a source of long-term exposure to arsenic.

Two other review articles received an average of 16 citations per post-publication year. The paper published in the *International Journal of Food Microbiology* in 2004 covered state of the art research on foodborne viruses (15). Norovirus and hepatitis A virus are highly infectious and lead to wide spread outbreaks of disease because they can persist in food manually handled by an infected food-handler and if such food is not heated or treated in other way after handling. Thus, greatest attention in preventive efforts should be given to good manufacturing practice to avoid introduction of viruses during food handling. The article on the control of *Listeria monocytogenes* (16) in the food-processing environment was published in the *Journal of Food Protection* in 2002, addressing the problem of large outbreaks of scattered cases after a virulent strain got established in the food-processing chain and thus infected multiple food lots over a short period of production. To increase the safety of ready-to-eat foods, there is a need to establish a sampling programme in the production environment, organization and interpretation of collected data and appropriate response to the positive finding of *Listeria* contamination.

The article with the highest citation rate (annual average of 44 citations) among all 8044 retrieved articles was published in *Nature* in 2005 (17), describing the genome sequencing and analysis of *Aspergillus oryzae*, a fungus used in the production of traditional fermented foods and drinks in Japan. The article shows that the genome of this *Aspergillus* species acquired specific expansion of genes for secretory hydrolytic enzymes, amino-acid metabolism and amino-acid/sugar uptake transporters, making it a suitable organism for fermentation.

The article ranked as the 3rd among the 10 top publications came from a collaborative group of researchers in Japan and UK and explored the transporters of arsenite in rice plants (18). The article was published in the *Proceedings of the National Academy of Sciences USA* in 2008 and received an average of 16 citations each year since then. The authors demonstrated that the possible accumulation of carcinogenic arsenite in rice grains, which caused massive poisoning in some Asian countries, was due to two different types of transporters in the rice roots, which are also used for silicone transport. High expression of genes for these two transporters in rice leads to silicone accumulation, which increases yield production, but also increases arsenic accumulation in the grains. The authors suggest that increasing silicone availability in the soil may suppress arsenic accumulation in rice and thus alleviate potential risk of arsenic poisoning.

The next most cited article, published in *Nature* in 2010 (19) and receiving 21 citations in the same year, described the new methodology for the non-destructive and ultra-sensitive visualisation of single molecules on surfaces. The formation a monolayer of gold nanoparticles as a ‘smart dust’ over surfaces allowed the demonstration of pesticide residues on citrus fruits.

High citation rate was achieved by the article presenting a case study of using a specific kinetic model to describe thermal inactivation of microbial vegetative cells in the food (20). The article was published in the *International Journal of Food Microbiology* in 2002. Based on published studies on thermal inactivation of microbial agents, the author made a theoretical exploration with a new mathematical model to calculate the necessary time and temperature treatment to pasteurize or sterilize foods.

Research articles describing outbreaks of common food poisoning also reached the top list of citation-intense publications. An epidemiological study of trends in indigenous foodborne diseases and deaths in England and Wales in 1992 to 2000 was published in the *Gut* in 2002 (21), and attracted an annual average of 15.6 citations. The authors analyzed routinely available surveillance data, special survey data and hospital episode statistics to estimate the burden and trends of indigenous foodborne disease. Between 1992 and 2000, the burden of indigenous foodborne disease fell by 53%.The most important pathogens were campylobacter, salmonella, *Clostridium perfringens*, verocytotoxin-producing *Escherichia coli* O157 and *Listeria monocytogenes*. In 2000, campylobacter still remained the highest threat, and the control of other pathogens was required to lower the mortality rates. A description of prevalence of *Campylobacter species*, *Escherichia coli* and *Salmonella* serovars in retail meat products from Greater Washington DC area in the USA was published in the *Applied and Environmental Microbiology* in 2001 (22) and received an average of 13 citations annually since its publications. The authors analysed 825 samples of retail raw chicken, turkey, pork and beef meat from supermarkets, and found that retail raw meats were often contaminated with foodborne pathogens. Chicken meat was more contaminated with *Campylobacter* that any other meat (70% of samples in comparison to 14% in turkey and 1.7% in pork and 0.5% in beef). The authors called for the introduction of stricter measures for ensuring food safety, particular the implementation of hazard analysis of critical control points (HACCP), as well as increased consumer education efforts to ensure food safety at home.

Finally, an article on the toxicity of polybrominated diphenyl ethers (PBDE) in foodstuffs also reached high citation rate and was ranked the 9th on the top 10 list, with 15 citations per year since its publication in the *Journal of Agricultural and Food Chemistry* in 2003 (23). PBDE is used as a flame retardant and seems to be present in a number of food samples, mostly in meat products and eggs, with an estimated dietary intake for an adult male of 97 ng/d in an area in Spain.

Taking into consideration all limitations of a scientometric analysis of research topics (9-12), the most useful topics in food safety during the last decade, according to their impact in research community, seemed to have been food-borne pathogens and toxins. We have also witnessed the emergence of genetic studies and new sophisticated methodologies for detecting small amounts of toxin residues on surfaces. Epidemiological and survey studies showed that there was a systematic effort to document, rapidly detect and control epidemic spread of disease. Some of these measures decreased the threat to food safety in developed countries, but there is still much room for improvement. Novel areas for improving the safety of food in the developing countries were also opened, such as the study on the potential molecular targets to alleviate accumulation of arsenic in rice. The five keys to safer food of the WHO (3) will remain most relevant for the developed as much as the developing world. As in other areas of research and life, the human factor is the most important one for the safety of food, and cannot be fully replaced by a novel chemical, agricultural or processing technology or gene transfer.
